# Role of the Air-Blood Barrier Phenotype in Lung Oxygen Uptake and Control of Extravascular Water

**DOI:** 10.3389/fphys.2022.811129

**Published:** 2022-03-28

**Authors:** Giuseppe Miserocchi, Egidio Beretta, Ilaria Rivolta, Manuela Bartesaghi

**Affiliations:** Dipartimento di Medicina e Chirurgia, Università di Milano-Bicocca, Monza, Italy

**Keywords:** alveolar-capillary equilibration, hypoxia, exercise, alveolar diffusion, alveolar perfusion, shunt effect

## Abstract

The air blood barrier phenotype can be reasonably described by the ratio of lung capillary blood volume to the diffusion capacity of the alveolar membrane (*Vc/Dm*), which can be determined at rest in normoxia. The distribution of the *Vc/Dm* ratio in the population is normal; *Vc/Dm* shifts from ∼1, reflecting a higher number of alveoli of smaller radius, providing a high alveolar surface and a limited extension of the capillary network, to just opposite features on increasing *Vc/Dm* up to ∼6. We studied the kinetics of alveolar-capillary equilibration on exposure to edemagenic conditions (work at ∼60% maximum aerobic power) in hypoxia (HA) (P_I_O_2_ 90 mmHg), based on an estimate of time constant of equilibration (τ) and blood capillary transit time (*Tt*). A shunt-like effect was described for subjects having a high *Vc/Dm* ratio, reflecting a longer τ (>0.5 s) and a shorter *Tt* (<0.8 s) due to pulmonary vasoconstriction and a larger increase in cardiac output (>3-fold). The tendency to develop lung edema in edemagenic conditions (work in HA) was found to be directly proportional to the value of *Vc/Dm* as suggested by an estimate of the mechanical properties of the respiratory system with the forced frequency oscillation technique.

## Introduction

It is a common experience that, in edemagenic conditions, inter-individual differences in the control of lung fluid balance are observed; the characteristic example is the proneness to develop lung edema on exposure to high altitude (Busch et al., 2001; Dehnert et al., 2006; Richalet et al., 2012, 2021; Eichstaedt et al., 2020a,b).

Finding the reason for these differences relating to the lung fluid balance has remained elusive for a long time. A line of research from our laboratory analyzed this problem and developed a project along the following lines of research:

(1) To determine the inter-individual differences in the morpho-functional features of the air-blood barrier in terms of membrane diffusion capacity (*Dm*) and extension of the capillary network from the estimate of capillary blood volume (*Vc*).
(2) To estimate the phenotype-dependent adaptive functional response of the air-blood barrier on exposure to edemagenic factors.
(3) To estimate the impact of points 1 and 2 on oxygen alveolar-capillary equilibration.

The results supported the hypothesis that the tendency to develop lung edema can be explained, considering a functional link between inborn features, perturbation in the capillary-to-interstitial fluid exchange, and the corresponding impact on the efficiency of gas exchange.

We shall, therefore, start this review by summarizing key principles of control of extravascular lung water as they represent the basis to understand the individual phenotype-dependent functional response to the edemagenic condition.

## The Control of Extravascular Water in the Air-Blood Barrier

The time course of the events leading to the development of lung edema has been described in a recent review (Beretta et al., 2021). We summarized here only the basic concepts useful to delineate the inter-individual differences in response to edemagenic conditions.

The very high surface area of the air-blood barrier (∼2,000 cm^2^/g) and its extreme thinness (∼0.1 μm in its thin portion) serve the gas diffusion function. The thinness of the air-blood barrier reflects a strict control of extravascular water volume that is kept at minimum, thanks to the extremely low water permeability across the endothelial and the epithelial barriers that strongly limit water fluxes. The water content of the lung is well defined by the wet weight to the dry weight ratio (W/D) that, in physiological conditions, is ∼5. Trans-capillary and trans-epithelial water exchanges (*J*_*v*_)are governed by the Starling law (Eq. 1), where *P* and Π are the hydraulic and the colloidosmotic pressures across any two compartments, *Kf*(*filtrationcoefficient*) = *Lp*⋅*A*, being *Lp* the water conductance, *A* the surface area available for flow, [(*P*_1_−*P*_2_)−σ(Π_1_−Π_2_)] is the Starling pressure gradient generating flows, σ being the protein reflection coefficient that defines the selectivity of the barriers to plasma proteins:


(1) $$J_{v}=K\,f\cdot \left[\left(P_{1}-P_{2}\right)-\sigma \,\left(\Pi_{1}-\Pi_{2}\right)\right]$$

A valid representative model of structure-function of the air-blood barrier rests on the comparison between oxygen diffusion that is in the range of 15 × 10^–2^ ml/(min cm^2^) in resting conditions, with capillary microvascular filtration that would be at least 10,000 less (Miserocchi, 2009); in other words, the air-blood barrier is very permeable to gases but minimally permeable to water. Edemagenic conditions include the increase in pulmonary blood flow, causing an increase in *Kf* due to the increase in both water conductance (*Lp*), the surface area of fluid exchange (*A*), as well as protein permeability (decrease in σ). The lung is normally well equipped to respond to increased microvascular filtration due to a specific morpho-functional feature, namely, the very low compliance of the interstitial structure, ∼0.5 ml mmHg^–1^⋅100 g of wet weight^–1^ (Miserocchi et al., 1993). The latter reflects the macromolecular organization of the proteoglycan component (Negrini et al., 1996; Miserocchi et al., 1999). In case of increased filtration in the interstitial space, water is captured by hyaluronan to form a gel, whose increase in steric hindrance causes a remarkable increase in interstitial pressure from ∼−10 cm H_2_O (physiological condition) up to ∼ +5 cm H_2_O (Miserocchi et al., 1993). Gel formation, as long as the filtration coefficient and the protein reflection coefficient remain within physiological values, provides a “*safety factor*” against edema formation as the increase in interstitial pressure buffers further filtration and may actually favor fluid reabsorption. To offset an increase in microvascular filtration rate, lymphatics can provide a passive negative-feedback control loop (Miserocchi, 2009). Lymph flow increases in proportion with the rate of increase in lung weight, which reflects the microvascular filtration (Roselli et al., 1984; Mitzner and Sylvester, 1986).

With the “*safety factor*” on, the water accumulation in the interstitial compartment is maintained within 10% of the control value; thus, the *W/D* ratio is kept at ∼ 5.5 (Miserocchi et al., 2001; Negrini et al., 2001). Inflammatory states (e.g., severe hypoxia, hyperoxia, surgery, excessive parenchymal stress/strain, and bacterial/viral infection) may cause severe damage to the native architecture of the proteoglycan family (Negrini et al., 1996; Miserocchi et al., 1999; Passi et al., 1999); the ensuing result is an uncontrolled increase of water and protein permeability. The critical phase of developing edema pivots on reaching a *W/D* of ∼ 6.5 (Beretta et al., 2021); modeling of this phase reveals an abrupt onset of edema with a short time constant (∼4–6 min) (Parker and Townsley, 2004; Mazzuca et al., 2016). Neither CT scan nor ultrasound can correlate with *W/D* ratios, corresponding to the early stages of perturbation in lung fluid balance before the condition becomes life-threatening.

## Inter-Individual Differences in Diffusion Lung Capacitance and Microvascular Perfusion of the Air-Blood Barrier

Wide inter-individual differences have been reported for alveolar oxygen uptake, even after normalizing diffusive parameters to individual total lung volume (Hughes and Pride, 2001). A breakthrough to interpret these differences came from the measurement of *DO*_2_, *Dm*, and *Vc* (oxygen diffusion capacitance, membrane diffusive capacitance, and capillary blood volume, respectively, Roughton and Forster, 1957) at different lung volumes from functional residual volume up to total lung volume. The lung volume dependence of these parameters allows delineation of the individual morpho-functional features of the air-blood barrier and to relate the differences in oxygen uptake and transport to match oxygen requirement, reflecting the individual phenotype (Miserocchi et al., 2008).

**FIGURE 1:**
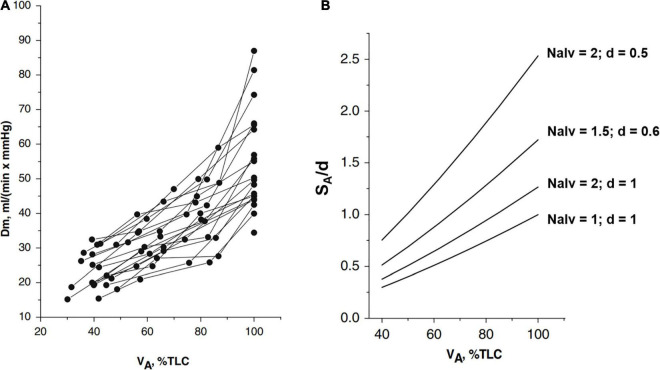
**(A)** Individual plots of *Dm* (a membrane diffusing capacity) vs. lung volume (*V*_*A*_) from *FRC* (about 40% *TLC*) up to 100% *TLC*. **(B)** Numerical simulation to show the lung volume dependence of surface to the thickness ratio of the air-blood barrier (*S_*A*_/d*). The various relationships were obtained by changing *Nalv* (number of alveoli) that affects *S_*A*_/d*, as indicated; for the reference condition, *S*_*A*_, *Nalv*, and *d* are set to unity (from Miserocchi et al., 2008).

Figure 1A shows that the increase in *Dm* on increasing lung volume remarkably differs among subjects. The highest *Dm* values at total lung capacity (*TLC*) were found in subjects displaying the highest increase in *Dm* on increasing lung volume. These differences have been interpreted, considering that *Dm* is proportional to $\frac{S_{A}}{d}$, *S*_*A*_ being the overall surface of the air-blood barrier and *d* its thickness. The decrease in *d* of the air–blood barrier on increasing lung volume was calculated as *1/S_*A*_*, considering the air-blood barrier as a lamina of constant volume (for details of the computational model, refer to Miserocchi et al., 2008). The simple geometrical reasoning is that lung diffusion is proportional to the alveolar surface; for a given lung volume, a greater increase in the lung surface on increasing lung volume is expected the higher the number of alveoli. A numerical simulation (Figure 1B) allows estimation of the dependence of $\frac{S_{A}}{d}$on lung volume by considering different phenotypes having different numbers of alveoli *Nalv* and/or different values of *d*, as specified in the figure. One can appreciate that a ∼3-fold difference (say from 0.25 to 0.75) in SA/d at Functional Residual Capacity (FRC) may justify a similar difference found in *Dm* on increasing lung volume up to *TLC* (Panel A). Accordingly, an inter-individual difference in alveolar number and thickness of the air-blood barrier can justify a corresponding difference in *Dm.* Regardless of an individual number of alveoli, we may recall that (Miserocchi et al., 2008), up to a volume of ∼70% TLC, most of the increase in $\frac{S_{A}}{d}$ is due to the increase in S_A_; above this volume, the increase in $\frac{S_{A}}{d}$ mostly reflects the decrease in *d* (the unfolding/folding zone, Beretta et al., 2021).

**FIGURE 2:**
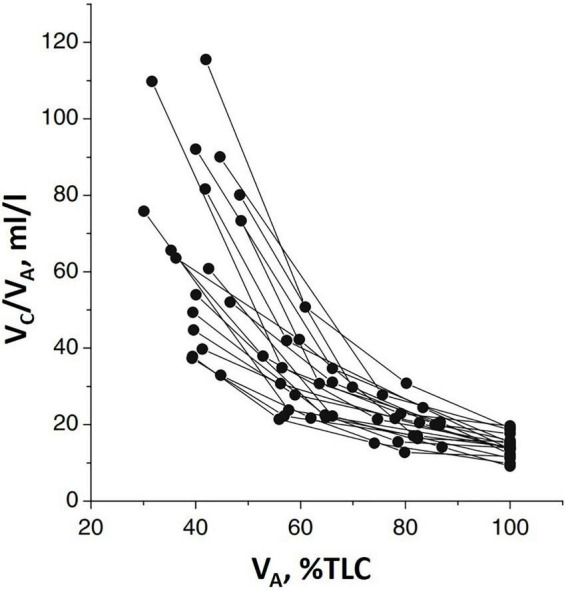
Individual plots of the ratio of pulmonary capillary blood volume (*Vc*) vs. lung volume (*V*_*A*_) as % of total lung capacity *V*_*A*_ (% *TLC*) (from Miserocchi et al., 2008).

Figure 2 shows that *Vc* (normalized to lung volume *V*_*A*_) decreases on increasing lung volume (as *% TLC*) due to the parenchymal stretching, squeezing the pulmonary capillaries and, thus, reducing their patency (Glazier et al., 1969; Mazzone et al., 1978; Brower et al., 1990; Koyama and Hildebrandt, 1991). Large inter-individual differences of *Vc* were also found; higher values at *FRC* suggest a greater extension of the alveolar-capillary network. Furthermore, the higher the *Vc* value at *FRC*, the greater its decease in increasing lung volume.

**FIGURE 3:**
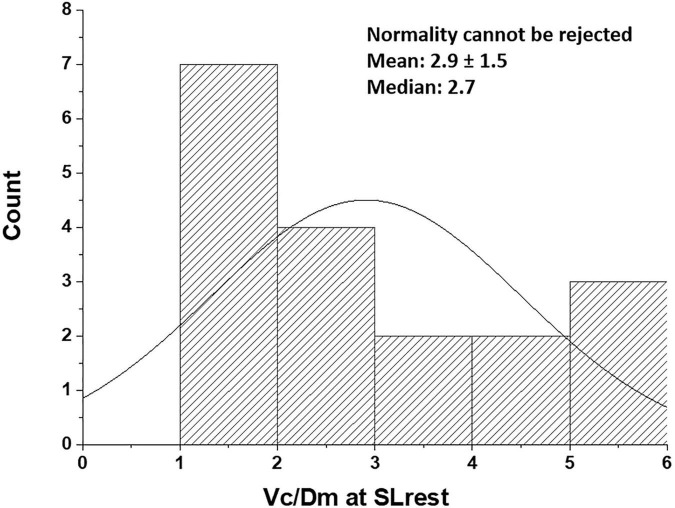
Distribution of *Vc/Dm* in the population studied at the sea level rest at 100% *TLC* [data from Bartesaghi et al. (2014)]. Values are expressed as ml/(ml ⋅min^–1^ ⋅mmHg^–1^).

We rely on the *Vc/Dm* ratio to identify the differences in the phenotype of the air-blood barrier to derive indications on the geometry of the alveoli and the extension of the capillary network. Note that, at *FRC*, this ratio would be mostly affected by the value of *Vc*, while, at 100% *TLC*, the ratio would be most affected by the increase in *Dm*. Since lung diffusion and subcomponents are routinely measured at 100% *TLC*, we present in Figure 3 the distribution of *Vc/Dm*, referring to 100% *TLC* at sea level (SL) at rest that appears to be normal (Shapiro-Wilk test, Orgin pro-2020; at the 0.05% level, the data were significantly drawn from a normally distributed population). The coefficient of variation for repeated intra-subject measurements did not exceed 12%, while, on pooled data, the coefficient of variation approached 50%, confirming the inter-individual differences.

Thus, subjects with low *Vc/Dm* on the left tail have a less developed capillary network and a relatively high number of small alveoli, providing a high surface of the air-blood barrier, while subjects with high *Vc/Dm* on the right tail have a more extended capillary network and a lower number of larger alveoli.

## Inter-Individual Differences in Vasomotion in Edemagenic Conditions

Given the differences in the air-blood barrier phenotype based on the *Vc/Dm* ratio, a reasonable question was to estimate how edemagenic conditions would affect pulmonary vasomotion in the capillary bed. The experimental model has shown that precapillary vasoconstriction involves vessels with a diameter of about 80 μm (Negrini et al., 2001). The question appeared justified, considering that subjects with a high *Vc/Dm* ratio would be more exposed to edemagenic conditions, being endowed with a more extended alveolar capillary network and, thus, a greater overall capillary surface (*A*). Subjects were studied at rest and in various conditions, implying exposure to edemagenic factors, namely, work at an SL, hypobaric HA at rest, and during work [∼ 60% maximum aerobic power at two heights (3,269 m, P_I_O_2_ 107 mmHg and at 3,840 m, P_I_O_2_90 mmHg)]. Work represents an edemagenic factor as it implies increased lung blood flow (McKenzie et al., 2005; Hodges et al., 2007), and HA is a well-known potent factor causing an increase in microvascular permeability to water and solutes (Hansen et al., 1994; Dehler et al., 2006).

On exposure to edemagenic factors, remarkable de-recruitment of pulmonary capillaries was found in the subjects with high *Vc/Dm* while minor-derecruitment or some recruitment was documented in the subjects with low *Vc/Dm* (Bartesaghi et al., 2014; Beretta et al., 2017). The mechanical properties of the respiratory system were also determined on hypoxia exposure with the forced frequency oscillation technique; results showed that, relative to the SL at rest, the respiratory reactance decreased to a greater extent in the subjects with high *Vc/Dm;* furthermore, in the same subjects, a 4-fold increase in the frequency dependence of respiratory resistance was found (Bartesaghi et al., 2014). Both results may be considered as indexes of greater perturbation of lung fluid balance (Dellacà et al., 2008).

The effect of pulmonary precapillary vasomotion should be considered specifically in relation to the change in water permeability (*Lp*). In case *Lp* remains unmodified, capillary recruitment favors gas diffusion by increasing the capillary gas exchange surface area (*A*) and the pool of hemoglobin to bind oxygen. On the other hand, if *Lp* is increased, capillary recruitment would lead to a remarkable increase in *Kf* due to the multiplicative effect of *Lp* ⋅ *A* (Mazzuca et al., 2016). As stressed in the recent paper (Beretta et al., 2021), massive filtration may occur down a large increase in *Kf* but a small Starling driving pressure gradient.

The advantage of capillary recruitment seems to prevail in the subjects with low *Vc/Dm* while, in the high *Vc/Dm* subjects, the disadvantage may justify the capillary de-recruitment.

A computational model of a morphologically based alveolar-capillary unit showed that, besides precapillary vasoconstriction, a further mechanism contributes to capillary de-recruitment. This resides in the compressive effect of positive interstitial pressure acting on the capillary surface during edema formation (Mazzuca et al., 2016). This phenomenon may also well occur in humans as the administration of a vasodilator agent cannot restore blood flow in edematous lung regions (Scherrer et al., 1996). In the presence of capillary derecruitment, blood flow is directed toward nonedematous regions and corner vessels (Koyama et al., 1989; Rivolta et al., 2011; Mazzuca et al., 2019). Interestingly, in unperfused capillaries, fluid reabsorption from the interstitial compartment may occur due to a decrease in capillary hydraulic pressure, thus, favoring recovery from edema (Kurbel et al., 2001).

**FIGURE 4:**
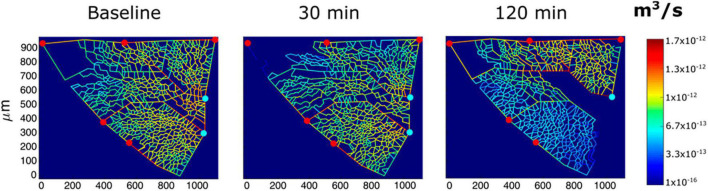
Results from modeling of alveolar perfusion in edemagenic condition (12% O_2_ exposure). Red and light blue dots identify, respectively, arteriolar accesses and venular exits. The color panels show capillary blood flow rates at different time points (baseline, 30; and 120 min) with color-coded log-scale intensity. In regions where edema develops, capillary blood flow is progressively reduced, approaching zero (from Mazzuca et al., 2019).

*In vivo* imaging data from an experimental model were also used to derive semi-quantitative estimates of the role of vasomotion in the control of blood flow and microvascular filtration (Mazzuca et al., 2019). Based on the model developed by Mazzuca et al. (2016), the results indicated that in alveolar units with larger alveoli and a greater extension of the septal network, microvascular filtration flow was greater on exposure to HA, as indicated by the increase in thickness of the interstitial space, and in these units, blood flow limitation increased over time. This can be appreciated in Figure 4, showing a 2D image-based model of the decrease in capillary blood flow as a change in color from yellow to blue (Mazzuca et al., 2019) in regions becoming edematous on exposure to hypoxia (12% of O_2_ balanced nitrogen). The model also showed that flow limitation in the alveolar-capillary network caused greater perfusion of alveolar corner vessels.

From the experimental model to humans, the point can be made that the subjects having a high *Vc/Dm* ratio appear to be endowed with larger alveoli compared to the subjects with a low *Vc/Dm* ratio and, thus, for this reason, more exposed to the risk of edema. Precapillary vasoconstriction has been reported as the reflex response to stimulation of interstitial vagal “*J*” (juxta-capillary) receptors whose afferent discharge was found to increase in exposure to edemagenic factors (Paintal, 1969).

It appears, therefore, tempting to hypothesize that precapillary vasoconstriction in the high *Vc/Dm* subjects represents a functional response aimed at limiting microvascular filtration to prevent/attenuate edema formation in edemagenic conditions. Inborn differences in microvascular permeability may also be invoked to justify differences in a tendency to develop edema.

Notably, the subjects more prone to develop lung edema in HA have a greater increase in pulmonary arterial pressure. In this respect, the clinical overlap of high-altitude pulmonary edema and pulmonary arterial hypertension has been recently discussed in terms of genetic background (Sharma et al., 2014; Eichstaedt et al., 2020a,b). Interestingly, the opposite behavior concerning lung vasomotion in hypoxia was also described for the systemic circulation. Indeed, in high-altitude pulmonary edema-susceptible (HAPE-S) mountaineers, a decrease in forearm blood flow was found on HA exposure, unlike in non HAPE-S subjects. This finding was attributed to impaired vascular endothelial function due to decreased bioavailability of NO (Berger et al., 2005). A decrease in exhaled NO was also found in HAPE-S subjects on exposure to normobaric hypoxia (Busch et al., 2001), as well as in patients with HAPE (Duplain et al., 2000). On a causative basis, it remains to be established whether the low bioavailability of NO depends on impairment of the biochemical pathway or, conversely, represents the functional response to counteract edema formation.

## Inter-Individual Differences in the Kinetics of Alveolar-Capillary Equilibration

The venous admixture, which includes the mismatch of ventilation to blood perfusion, ${\dot{V}}_{A}/\dot{Q}$ (Domino et al., 1993), and shunt (Stickland et al., 2004; Lovering et al., 2006, 2008) are well-known causes leading to incomplete alveolar-capillary oxygen equilibration. We present here the relevance of a shunt-like effect, depending on the transit time in the pulmonary capillaries that reflect the increase in a cardiac output and the individual control of lung vasomotion in edemagenic conditions. A valid model defining the alveolar-capillary equilibration across the air-blood barrier (Piiper and Scheid, 1981) has been presented based on a mass balance equation. Defining$d\,\dot{M,}$ the oxygen mass transport across the air-blood barrier; $\dot{Q,}$ the cardiac output; and *dC*, the increase in blood oxygen concentration along the length of the pulmonary capillary (*x*), the following equation holds:


(2) $$d\,\dot{M}\,\left(x\right)=\dot{Q}\cdot d\,C\,\left(x\right)$$

The mathematical development of Equation 2 allows description of an exponential increase of *dC*(*x*) to reach an equilibrium at the exit of the pulmonary capillary (*Leq*) given by:


(3) $$L\,e\,q=e^{-\frac{D\,O_{2}}{\beta \,\bar{Q}}},$$

where *DO*_2_ is the O_2_ diffusive capacity and β is the Hb-binding capacity for O_2_.

A development of this model allows the definition of the equilibration process as a function of time (*t*) as blood flows along the capillary, thus:


(4) $$d\,\dot{M}\,\left(t\right)=\dot{Q}\cdot d\,C\,\left(t\right)$$

Based on Equation 4, the equilibrium at the exit from the capillary may be written as (Beretta et al., 2019):


(5) $$L_{e\,q}=e^{-\frac{T\,t}{\tau }},$$

Being *Tt* the blood transit time in the pulmonary capillary estimated as the ratio of the lung capillary volume (*Vc*) to a cardiac output ($\dot{Q}$):


(6) $$T\,t=\frac{V\,c}{\dot{Q}}$$

and the time constant of the equilibration process is defined as:


(7) $$\tau =\frac{\beta \,V\,c}{D\,O_{2}}$$

At the exit from the pulmonary capillary, the value of *Leq* is the same from Equations 3 and 5. The *Leq* can vary from 0 (the case of perfect equilibration) to 1 (the case of 100% shunt).

Equation 5 allows the definition of the time course of the equilibration process in response to increased oxygen demand based on the blood transit time in the pulmonary capillary, resulting from the interaction between the increase in cardiac output and the available lung capillary network.

**FIGURE 5:**
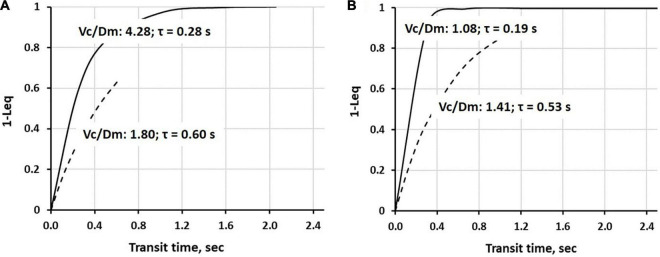
Time course of 1-*Leq* (an index of alveolar-capillary equilibration) in normoxia at rest (a continuous line) and on exposure to severe hypoxia (3,840 m, P_I_O_2_90 mmHg, a dashed line) in two representative subjects having a high **(A)** or a low **(B)** *Vc/Dm* ratio at the sea level (SL) in resting condition (data from Beretta et al., 2019).

Continuous lines in Figure 5 show the time course of alveolar-capillary equilibration for the two subjects at rest in normoxia *Vc/Dm* of 4.28 (Panel A) and 1.08 (Panel B), respectively. For the sake of graphical representation, we put on the ordinate 1-*Leq*, meaning that the case of perfect equilibration implies *Leq* = 1. In normoxia at rest, equilibration kinetics were remarkably slower in the subjects with a high *Vc/Dm* due to a correspondingly longer time constant (Eq. 7); in both subjects, *Tt* was long enough to allow complete equilibration. During work in severe HA (3,840 m, P_I_O_2_90 mmHg, dashed lines) in both subjects, the time constant was increased, slowing down the kinetics of equilibration. However, the remarkable shortening of *Tt* (Panel A), reflecting precapillary vasoconstriction, limited the equilibration at 0.6, while, in Panel B, equilibration was only slightly decreased due to a longer *Tt*. Thus, during work performed in hypoxia, facing an average P_*A*_O_2_ ∼ 55 mmHg (Beretta et al., 2017), some individuals can still reach a satisfactory alveolar-capillary equilibration, while, in other subjects, this process may be strongly limited by precapillary vasoconstriction (Mazzuca et al., 2016; see also Figure 4).

**FIGURE 6:**
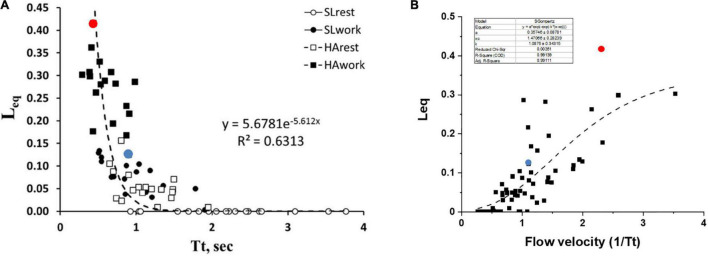
**(A)** Correlation between transit time (*Tt*) and *Leq* in various conditions as indicated in the inset. **(B)** A plot of *Leq* vs. *1/Tt* (an index of blood velocity). Red and blue dots refer to the two subjects shown in Figures 5A,B, respectively (data from Beretta et al., 2019).

Figure 6A shows the pooled data of *Leq* vs. *Tt* in various conditions, as indicated by the legend. It appears that *Leq* remains at zero (complete alveolar-capillary equilibration for O_2_) as long as *Tt* is greater than ∼1.5 s, while it increases exponentially for Tt <1.5 s. Of course, we refer to an average value of *Tt* along the pulmonary vascular tree, although a regional dispersion of this index has been reported (Capen et al., 1990; Clough et al., 1998).

One may further consider that blood flow velocity in the alveolar capillaries can be expressed as: *Vel*∝*1/Tt*; Figure 6B shows the values of *Leq* plotted vs. *1/Tt*.

The shear rate at the endothelial capillary wall is a recognized cause of the increase in microvascular and protein permeability and is expected to increase with increasing blood flow velocity (Sill et al., 1995; Lakshminarayanan et al., 2000; Kang et al., 2014). Looking at the dispersion of the data in Figure 6B, one shall comment that the balance between an anti-edemagenic response (precapillary vasoconstriction) and its inevitable edemagenic consequence (the increase in the shear rate) might vary among individuals. One can hypothesize that, for a given value of *1/Tt*, the prevalence of the shear-dependent increase in permeability may justify a greater value of *Leq* due to some degree of interstitial fluid accumulation. This may be the case for the subjects with high *Vc/Dm* (a red dot, Figure 6B) as opposed to the subjects with low *Vc/Dm* (a blue dot, Figure 6B).

**FIGURE 7:**
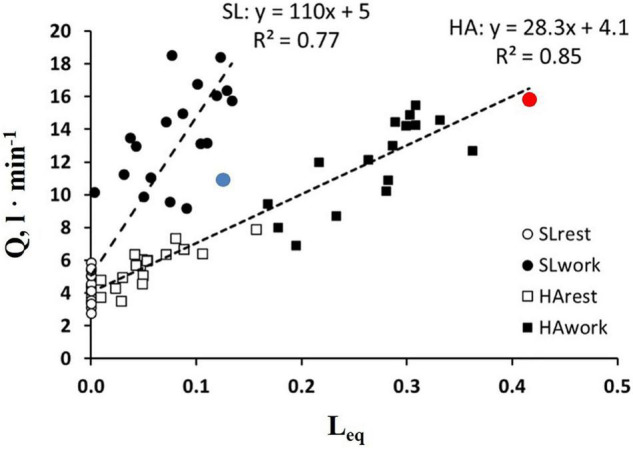
Correlation between *Leq* and a cardiac output ($\dot{Q}$) in the condition indicated. Red and blue dots refer to the two subjects shown in Figures 5A,B, respectively (from Beretta et al., 2019).

It should be considered that *Tt* reflects both a local phenomenon relating to vasomotion as well as the increase in cardiac output (Eq. 6). The latter varied remarkably among subjects. The impact of the increase in the cardiac output on *Leq* can be appreciated in Figure 7, showing the relationships between the cardiac output vs. *Leq* in the same conditions reported in Figure 6A. The remarkable shift to the right of the relationship referring to work in normoxia at an SL to work in HA reflects the effect of precapillary vasoconstriction for a given cardiac output. During work in hypoxia at a similar percentage of oxygen consumption (relative to maximum), the cardiac output (normalized to body weight) was ∼50% greater in the subjects with high *Vc/Dm* (a red dot) compared to the subjects with low *Vc/Dm* (a blue dot).

**FIGURE 8:**
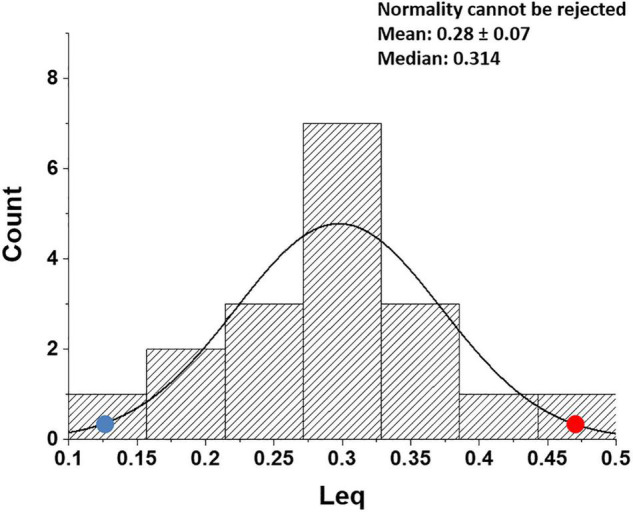
Distribution of *Leq* values in all subjects at the end of exercise in severe hypoxia (HA) (3,840 m, P_I_O_2_ 90 mmHg). Red and blue dots refer to the two subjects shown in Figures 5A,B, respectively (data from Beretta et al., 2019).

Figure 8 shows that, in healthy people, in the most edemagenic conditions (work in severe HA, P_*A*_O_2_ ∼ 55 mmHg), the distribution of *Leq* is normal. The positions of the two subjects referring to Figure 5 within the distribution (red and blue dots) reflect the inter-individual variability.

## Summary

The conceptual contribution of the research studies referred to in this review may be summarized as follows:

1. The air-blood barrier phenotype can be described by the distribution of the *Vc/Dm* ratio. *Vc/Dm* shifts from ∼1, reflecting a higher number of alveoli of smaller radius, providing a high alveolar surface and a limited extension of the capillary network, to opposite features for *Vc/Dm* increasing up to ∼ 4.
2. Differences in air-blood barrier phenotype impact the efficiency in gas exchange and control of extravascular lung water when facing an increase in oxygen demand in edemagenic conditions. A lower *Vc/Dm* appears to be more efficient to guarantee gas exchange, as predicted by a theoretical morpho-functional model (Sapoval et al., 2002). There are indications that a lower*/Dm* is more protective against the risk of lung edema; conversely, a high *Vc/Dm* implies a greater tendency to develop lung edema.
3. A shunt-like effect can be described based on capillary blood kinetics that reflects the individual lung vasomotor control and the increase in cardiac output. This effect is minimal for low *Vc/Dm*, while it may remarkably increase in the subjects with high *Vc/Dm* due to increasing lung capillary blood velocity, reflecting precapillary vasoconstriction and a greater increase in cardiac output.
4. The data confirm that the lung response to an edemagenic condition is functionally aimed at protecting the air blood barrier to avoid a perturbation of fluid balance.

## Conclusion

As far as we know, the present studies are the first ones of this nature, and we think they may provide a valuable contribution in terms of “*human integrative and translational physiology across a range of applied contexts, including exercise and environmental.”* Studies were performed in healthy subjects; accordingly, there is a potential interest to consider people reaching high altitudes on trekking expeditions being exposed to the risk of HAPE. Potential clinical relevance may also be considered as cardio-pulmonary disorders as well as conditions of decrease in vascular bed (lung resection and thrombosis) are at risk of developing lung edema. Defining the *Vc/Dm* ratio through ambulatory pneumological evaluation may turn useful to define the patient’s tendency to develop edema before an acute severe disease occurs.

From an operational point of view, the subject’s functional evaluation requires:

- The estimate of *Vc/Dm* at 100% *TLC*, relying on the *DLNO*/*DLCO* technique, at the SL at rest, and on exercise.
- The estimate of *Tt* that requires the measurement of cardiac output (by echocardiography with a semi-recumbent set-up at rest and on exercise).
- A valid potentiation of the trial requires the same determinations on exposure to normobaric HA.

## Data Availability Statement

The original contributions presented in the study are included in the article/supplementary material, further inquiries can be directed to the corresponding author.

## Author Contributions

GM conceived the research project and wrote the manuscript. EB, IR, and MB contributed to the discussion and analysis. All authors contributed to the article and approved the submitted version.

## Conflict of Interest

The authors declare that the research was conducted in the absence of any commercial or financial relationships that could be construed as a potential conflict of interest.

## Publisher’s Note

All claims expressed in this article are solely those of the authors and do not necessarily represent those of their affiliated organizations, or those of the publisher, the editors and the reviewers. Any product that may be evaluated in this article, or claim that may be made by its manufacturer, is not guaranteed or endorsed by the publisher.
